# Protocol for Shoulder function training reducing musculoskeletal pain in shoulder and neck: a randomized controlled trial

**DOI:** 10.1186/1471-2474-12-14

**Published:** 2011-01-14

**Authors:** Christoffer H Andersen, Lars L Andersen, Ole S Mortensen, Mette K Zebis, Gisela Sjøgaard

**Affiliations:** 1 National Research Centre for the Working Environment, Lersø Parkalle 105, DK 2100 Copenhagen Ø, Denmark; 2 Department of Occupational and Environmental Medicine, Bispebjerg University Hospital, DK 2400 Cph NV, Denmark; 3 Institute of Sports Science and Clinical Biomechanics, University of Southern Denmark, DK 5230 Odense M, Denmark

## Abstract

**Background:**

Neck and shoulder complaints are common among employees in sedentary occupations characterized by intensive computer use. Such musculoskeletal pain - which is often associated with restricted range of motion and loss of muscle strength - is one of the most common conditions treated by physical therapists. The exact mechanism of neck pain is rarely revealed by clinical examination and the treatment has varied from passive rest to active treatments. Active treatments have often been divided into either training of the painful area or the surrounding musculature avoiding direct training of the painful area. Our study investigates the effect of the latter approach.

**Methods/Design:**

A randomized controlled trial of 10 weeks duration is currently being conducted. Employed office workers with severe neck-shoulder pain are randomized to 3 × 20 min shoulder function training with training supervision or to a reference group receiving advice to stay physically active. Shoulder function training primarily focuses on the serratus anterior and lower trapezius muscle with only minimal activation the upper trapezius.

An announcement was sent to the administrative section of the university including jobs characterized by intensive computer work. The first 100 positive replies entered the study. Among these inclusion criteria were pain intensity in the neck/shoulder of at least 3 on a 0-9 scale. Exclusion criteria were cardiovascular disease, trauma, hypertension, or serious chronic disease. Before and after the intervention period the participants replied to a questionnaire about musculoskeletal disorders and work disability, and underwent a standardized clinical examination of the neck and shoulder girdle. Further, on a weekly basis the participants log pain intensity of the neck and shoulder during the previous week.

The primary outcome measure is pain in the neck and shoulders at week 10 based on the weekly pain registration and results from the clinical examination. Secondary outcomes are strength and work disability.

**Trial Registration:**

ClinicalTrials (NCT): NCT01205542

## Background

Neck and shoulder complaints are common among employees in sedentary occupations characterized by intensive computer use[1]. Chronic neck pain is often a widespread sensation with hyperalgesia in the ligaments and muscles during both passive and active movements. Musculoskeletal pain - which is associated with restricted range of motion and functional loss[2] - is one of the most common conditions treated by physical therapists. The occurrence of neck/shoulder muscle pain has increased during recent decades and computer work is particularly associated with neck symptoms[3,4].

Physical exercise is a cornerstone in health and well-being[5]. An increasing number of studies and reviews within the last decade provide evidence for the effectiveness of physical exercise at the workplace in managing musculoskeletal pain[6-8]. The origin of chronic neck pain is multifactorial, including physical strain and psychosocial stress[9]. Treatment regimes has varied from complete rest to high-intensity strength training [10,11]. Excess activation of the upper trapezius, combined with decreased control of the lower trapezius and the serratus anterior likely contributes to neck/shoulder pain. Supporting this, patients with shoulder disorders show an altered muscle activation balance towards increased upper trapezius activation and reduced serratus anterior activation [12]. Targeted rehabilitation of these types of neck/shoulder dysfunctions requires detailed knowledge of exercise-specific activation balance of the scapular muscles. Thus, restoration of balanced muscle activation is a challenge in the rehabilitation training. This study investigates in a randomized controlled design the efficiency of 10 weeks shoulder function training - i.e specific strength training of the lower trapezius and the serratus anterior muscle while minimizing direct training of the upper trapezius. We hypothesize that such exercise relieves neck/shoulder pain among office workers.

## Methods and design

### Study design

We are currently conducting a randomized controlled trial in Denmark. The trial duration is September 2010 to December 2010. The participants were recruited from a large university.

All of the participants gave their written consent to participate in the study. The local ethics committee approved the study protocol (H-C-2008-103), which qualified for registration in the ClinicalTrials.gov, number NCT01205542

### Study population

An announcement with a short introduction and invitation text, together with a link to an internet-based questionnaire was send to office workers from the administrative section of the university. When 100 had replied positive regarding participation to the questionnaire we closed for further recruitment based on a priori power calculations and drop out estimates. Out of the 100 responders 8 subsequently declined to participate in the study.

Table 1 shows the 100 responders with regard to age, body mass index, neck/shoulder pain and days with neck/shoulder pain within the last 12 months.

**Table 1:** Characteristics of the 100 employees responding to the questionnaire.

	All	Men	Women
N	100	26	74
Age	44 (11)	47 (13)	43 (11)
Height	171 (9)	180 (7)	168 (7)
Weight	72 (14)	83 (14)	68 (13)
BMI	24 (4)	25 (3)	24 (4)
Neck & shoulder Pain last month (0-9)	5.3 (2.2)	5.5 (2.3)	5.3 (2.1)
Days with neck & shoulder pain within the last 12 months	174 (138)	173 (134)	176 (150)

Inclusion criteria were pain intensity in the neck/shoulder of at least 3 on a 0-9 scale.

Exclusion criteria were a) hypertension (Systolic BP > 160, diastolic BP > 100) or cardiovascular diseases (e.g. chest pain during physical exercise, heart failure, myocardial infarction and stroke), b) symptomatic herniated disc or severe disorders of the cervical spine, c) postoperative conditions in the neck and shoulder region, d) history of severe trauma, and e) pregnancy, f) other serious disease.

**Figure 1:**
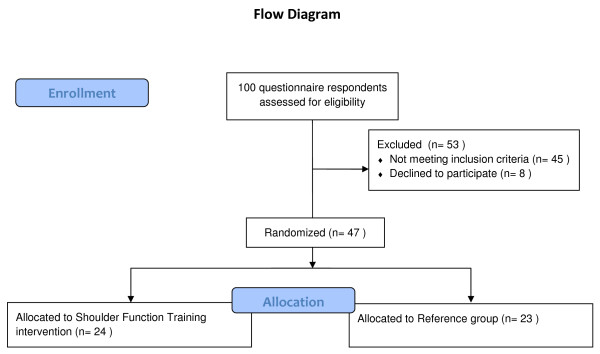
**Flow chart**.

Using a computer generated random numbers table, the remaining 47 participants were randomly allocated to training or control. Gender and age was used as stratification variables. Subsequently, participants were informed via email about group allocation. A flow-chart of the process is shown in Figure 1. Table 2 shows the baseline characteristics of the included participants.

**Table 2:** Characteristics of employees randomized into the two intervention groups.

	All	Reference	Training	P
N	47	23	24	
Women	37	18	19	
Men	10	5	5	
Age	44 (12)	45 (11)	44 (13)	0.86
Height	171 (7)	171 (8)	171 (7)	0.84
Weight	72 (12)	72 (12)	72 (13)	0.99
BMI	25 (4)	25 (4)	24 (3)	0.85
Neck & shoulder Pain last month (0-9)	5.6 (1.7)	5.4 (1.5)	5.7 (1.9)	0.64
Days with neck & shoulder pain within the last 12 months	212 (119)	211 (126)	213 (115)	0.96

### The intervention program

The training-group was allocated a total of one hour training per week for 10 weeks during working-hours. Experienced instructors assist all of the training sessions. The reference group was not offered any physical training, but encouraged to stay active, and they replied to the same questionnaires as the training-group.

**Figure 2:**
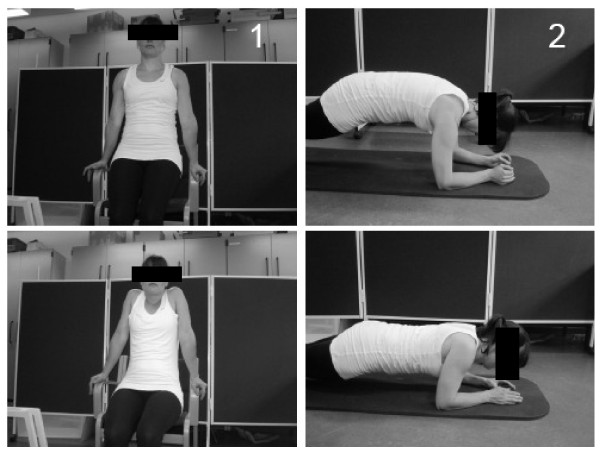
**The two main shoulder function exercises: 1) press-up, 2) push-up plus**.

The training-group performed shoulder function training with exercises which have shown to activate the serratus anterior and lower trapezius muscles to a high extend but with only a low activation of the upper trapezius. The exercises used have been selected on the basis of a yet unpublished study and the two main exercises -push-up plus and press-ups - are pictured in Figure 2. During the 10-week intervention training loads were progressively increased according to the principle of periodization and progressive overload. Each training session started with a short warm-up by slowly moving the neck, upper back, shoulder blades and shoulder joint through pain-free range of motion.

The participants received a training diary for registering training sessions. We encouraged participants to train with an instructor during a specified time period together with their colleagues. Instructors taught the participants how to perform the exercises, and helped with exercise adjustment when needed. The instructors focused on positive feedback to maintain motivation.

### Self-reported measures

We applied an email-based questionnaire including e.g. the Standardized Nordic questionnaire for musculoskeletal disorders[13], stages of change[14], work productivity[15] and work disability [16]. The main questions are described in more detail below. Each week the participants received an email asking them "How intense was your worst pain in the neck-shoulder area during the last week on a 0-9 scale?" (0 means no complaints and 9 means pain as bad as it can be).

*Musculoskeletal pain symptoms *of the neck, shoulder, arm, hand, and back were evaluated using scales concerning both intensity and duration of symptoms. Participants replied to the questions "How many days have you had trouble in [body part] during the last three months?" (0 days; 1-7 days; 8-30 days; >30 days; everyday) for symptom duration, and "On average, how intense was your pain in [body part] during the last three months on a 0-9 scale?" (0 means no complaints and 9 means pain as bad as it can be) for symptom intensity. Answers to the question that concerned symptom duration were recoded as follows: 0 days = 0, 1-7 days = 4, 8-30 days = 19, >30 days = 60, everyday = 90[1]. Illustrations from the Nordic questionnaire defined the respective body regions[13]. Further, headache was evaluated using a questionnaire on intensity, duration, and frequency of headache during the previous month.

*Stages of change *in relation to physical activity was in this study assessed by a questionnaire originally presented by Marcus and colleagues in 1992[17]. Questions asked in the questionnaire were e.g. "As far as I'm concerned, I don't need to exercise regularly", "I really think I should work on getting started with a regular exercise program in the next 6 months", and "I have started exercising regularly within the last 6 months".

*Productivity *was rated on an 11-step ordinal scale: "How do you perceive your overall productivity the last 4 weeks?" The rating went from 0 (the worst a worker could do) to 10 (the best a worker in the same job could do)[15].

Participants rated *work disability *at baseline and follow-up by the work module of the Disability of the Arm, Shoulder and Hand questionnaire (DASH): "In the past week did you have any difficulty:" 1) "using your usual technique for your work?", 2) "doing your usual work because of arm, shoulder or hand pain?", 3) "doing your work as well as you would like?", 4) "spending your usual amount of time doing your work?". Participants replied on a 5-point Likert scale from "No difficulty" to "Unable". The DASH score was normalized on a scale of 0-100 (by adding the 4 values, dividing by 4, subtracting by 1, and multiplying by 25)[16].

### Clinical measurements

A physiotherapist performed a thorough clinical examination of the neck and shoulder girdle of the participants as previously described in detail[18]. This included examination of neck and shoulder mobility, soreness during palpation, muscle tightness, shoulder impingement (Neers test and Hawkins test), and measuring the position of the angulus inferior and angulus superior on the scapula[19].

### Objective measures

Testing of physical capacity was performed at baseline and 10-week follow-up in both groups.

*Pressure Pain Threshold (PPT) *was measured at 4 sites using a standardized procedure[20]. Muscle and bone sites to be examined were located by palpation. The following points were outlined: 1) upper trapezius, 2) lower trapezius, 3) sternum and, 4) tibialis anterior.

*Maximal muscle strength *was assessed by a maximum isometric shoulder protraction test and shoulder elevation test against a pair of strain gauge dynamometers at baseline and at the end of the intervention (10 weeks).

*Muscle activation *during strength testing was assessed through surface electromyography (EMG). Electrodes were placed according to SENIAM recommendations on the skin over the following muscles: 1) upper trapezius, 2) middle trapezius, 3) lower trapezius, 4) upper serratus anterior and, 5) lower serratus anterior.

### Statistics

The primary outcome is change in pain of the neck and shoulders at 10 weeks. Secondary outcomes include the other measures mentioned above. We will firstly analyze the data according to the intention-to-treat principle and secondly, per protocol. We will use repeated measures analysis of variance. The following null-hypothesis will be tested;

1) There is no difference between the training group and reference group for the change in neck/shoulder pain from baseline to week 10.

Power analyses performed prior to the study showed that - to reject the null-hypothesis of equality - we should include 20 participants per group (allowing for a 20% loss to follow-up) for 80% power to detect a change in pain of 1,5 on a 0-9 scale between groups.

## Competing interests

The authors declare that they have no competing interests.

## Authors' contributions

GS, MKZ, OSM, LLA and CHA were responsible for the research design. CHA drafted the paper, and all co-authors made significant contributions to drafting the protocol. All authors have read and approved the final manuscript.

## Pre-publication history

The pre-publication history for this paper can be accessed here:

http://www.biomedcentral.com/1471-2474/12/14/prepub
